# Physicians’ appraisal of parental concerns: a qualitative assessment

**DOI:** 10.3399/BJGP.2024.0554

**Published:** 2025-07-29

**Authors:** Laura Cuypers, Cato Dessers, Birgitte Schoenmakers, Jaan Toelen

**Affiliations:** 1 Faculty of Medicine, KU Leuven, Leuven, Belgium; 2 Department of Pediatrics, University Hospitals Leuven, Leuven, Belgium; 3 Department of Public Health and Primary Care, KU Leuven, Leuven, Belgium; 4 Leuven Child and Youth Institute, KU Leuven, Leuven, Belgium; 5 Department of Development and Regeneration, KU Leuven, Leuven, Belgium

**Keywords:** Clinical gut feeling, parental concern, physician-patient relations, qualitative research, focus groups

## Abstract

**Background:**

In clinical encounters with children and their parents, physicians rely on both analytical and non-analytical factors to assess the clinical problem. Research on clinical gut feeling has recognised this as a significant diagnostic factor, yet little is known about how physicians evaluate parental concerns.

**Aim:**

To investigate which parent- and physician-related factors influence a physician’s assessment of parental concerns.

**Design and setting:**

Nine qualitative semi-structured focus group interviews were conducted with 15 GPs and 15 paediatricians in Belgium between May and August 2022.

**Method:**

The interview transcripts were independently coded and analysed thematically using the constant comparative analysis method.

**Results:**

The factors that physicians use to assess parental concerns can be categorised into four groups: parent-related, physician-related, context-related, and child-related factors. Within each category, there are multiple determinants, with the most influential being having multiple children as a parent, the physician’s work experience, and disease severity.

**Conclusion:**

This study confirms some determining factors that have already been described in the literature, but it also identifies new determinants (for example, having multiple children as a parent and physician fatigue). Quantitative research could assess the extent to which the identified factors are involved in the assessment of gut feeling.

## How this fits in

In the paediatric context, parental concerns can influence a physician’s assessment and management of the case next to the more objective clinical findings. This study confirms existing determinants of this appraisal, but also identifies having multiple children as a parent and physician fatigue as important factors.

## Introduction

Identifying severe illness in children can be challenging, and any delay in diagnosis and subsequent treatment may lead to significant morbidity and even mortality. Within this context, parents have a pivotal role, as they are responsible for promptly seeking medical attention when their child falls ill.^1^ When physicians assess a patient, their diagnostic thought processes and decision making should be primarily rooted in clinical evidence. However, in addition to analytical reasoning, they also rely on non-analytical reasoning and intuition as integral components of their medical judgement.^2^ At times, healthcare providers may experience an innate sense that something is ‘amiss’, a universal and transcultural phenomenon referred to as a ‘gut feeling’.^3–7^ Although this intuitive sensation does not rely on explicit evidence, it has demonstrated diagnostic value, particularly in the context of diagnosing serious infections in children.^8^

Prior research has extensively examined the symptoms and signs in children that trigger this gut feeling among healthcare providers.^8–10^ Additionally, non-clinical factors such as parental concerns can significantly influence a physician’s gut feeling.^8,10,11^ Notably, if parents convey that the illness is ‘distinct from their previous experiences’, it exerts a substantial impact on the physician’s gut feeling.^8^ Furthermore, a physician’s own clinical experience, medical knowledge, contextual awareness, and the patient’s medical history also contribute to the formation of this intuitive sense.^2,4,8,12^

To comprehensively explore the factors influencing the gut feeling of physicians beyond the clinical presentation, a precise and valid conceptualisation of this phenomenon is imperative. Stolper *et al* conducted focus group interviews to establish valid descriptions of this concept.^6^ They delineated the concept of gut feeling into two distinct facets: the ‘sense of alarm’ and the ‘sense of reassurance’. The sense of alarm is characterised as: *‘an uneasy feeling perceived by a GP as he/she is concerned about a possible adverse outcome, even though specific indications are lacking’*.^6^ Conversely, the sense of reassurance is defined as: *‘a secure feeling perceived by a GP about the further management and course of a patient’s problem, even though the doctor may not be certain about the diagnosis’*.^6^

This study aimed to investigate the factors beyond the clinical presentation that influence how physicians evaluate parental concerns and, consequently, their own ‘sense of alarm’. The exact nature of these parental concerns and their effects on physicians have not been extensively studied. This research focuses on several factors that contribute to, to varying degrees, a physician’s gut feeling.

## Methods

### Study design

A qualitative and exploratory study design was employed to conduct focus group sessions involving paediatricians and GPs. This design was selected because the primary focus of this study was on the subjective assessments of participants and the underlying mechanisms that influence their gut feelings. Focus group interviews were chosen partly due to the relatively large number of participants (efficiency) and due to the fact that these enable deeper discourses through the group dynamics, with diverse perspectives and critical reflection (interaction and synergy).

### Sample

Belgian GPs and paediatricians were recruited from the alumni network of the Faculty of Medicine at KU Leuven through email invitations. A total of 18 paediatricians and 62 GPs were contacted. The recruitment was balanced for the male–female ratio (2.13 for paediatrics, 0.78 for general practice), age, and geographical distribution. Based on willingness to participate, 15 physicians from each group were included.^13^

The participating physicians, from all five Flemish provinces and ethnically diverse cities like Antwerp, ensured representation from both urban and rural areas, as well as various ethnic backgrounds.^14^ Participants were required to have a minimum of 5 years of professional experience to ensure adequate familiarity with situations involving gut feelings.

### Data collection

The focus group sessions were conducted using semi-structured interviews, with two to six participants per session. The interview guide was constructed based on previously published research data to ensure comprehensive coverage of relevant topics (see Supplementary Information S1). Prior to the interviews, participants provided informed consent and furnished information regarding their age, gender, years of work experience, marital status, number of children, their child’s/children’s age/ages, and whether they, their children, or their partners, had ever experienced serious illness necessitating hospitalisation or referral to a specialist. The interviews were conducted via Zoom between May and August 2022 and had an average duration of 1 hour. Interviewers recorded field notes encompassing observations, reflections, and ideas during and immediately after each session. The interviews were transcribed verbatim with pseudonymisation.

### Data analysis

Transcript coding was carried out independently by two researchers utilising a Qualitative Analysis Guide of Leuven (QUAGOL)-based framework.^15^ The initial step in the coding process involved open coding, during which data were summarised and conceptual constructs were formulated. These summaries were subsequently deliberated on within the research team to develop a preliminary coding framework. The labelling process was undertaken individually by the two researchers, with additional meetings convened to reconcile discrepancies and reach consensus on shared labels. The second step involved axial coding, where overarching categories were created that were applicable across all focus group interviews. The third step encompassed selective coding, wherein associations established during the first or second step were corroborated by scrutinising the categories and data that were included or excluded across interviews. This meticulous coding process culminated in the identification of four main thematic areas. Furthermore, a Q-analysis was conducted to quantitatively assess participants’ attitudes toward the identified factors, providing a structured measure of subjective perspectives.

## Results

### Sociodemographic characteristics of the participants

Sociodemographic data are shown in Table 1. In total, 30 physicians (15 paediatricians and 15 GPs) participated in nine interviews. The mean age of participants was 44 years (range 27–67). One-half of the physicians had a minimum of 18 years’ working experience. Four participants did not have children of their own.

**Table 1.** Sociodemographic data of the study participants

Characteristic	All participants (*N* = 30)	Paediatricians (*n* = 15)	**GPs (*n* = 15)**
**Gender, female, *n* (%)**	18 (60.0)	10 (66.7)	8 (53.3)
**Mean age, years (SD)**	44 (10.6)	41.5 (8.8)	46.5 (11.5)
**Marital status, *n* (%)**			
Married	25 (83.3)	14 (93.3)	11 (73.3)
Cohabiting	4 (13.3)	1 (6.7)	3 (20.0)
Single	1 (3.3)	0 (0.0)	1 (6.7)
**Median working experience, years (IQR**)	18 (16)	11 (15)	25 (18)
**Mean number of children, *n* (SD)**	2.3 (1.4)	2.3 (1.1)	2.3 (1.6)
**Mean age of children, years (SD)**	15.5 (9.5)	10.9 (8.8)	19.8 (8.2)
**Faced with serious illness, *n* (%)**	10 (33.3)	7 (46.7)	3 (20.0)

IQR = interquartile range. SD = standard deviation.

### Critical assessment and identification of the non-clinical determinants of ‘gut feeling’

**Figure 1.**
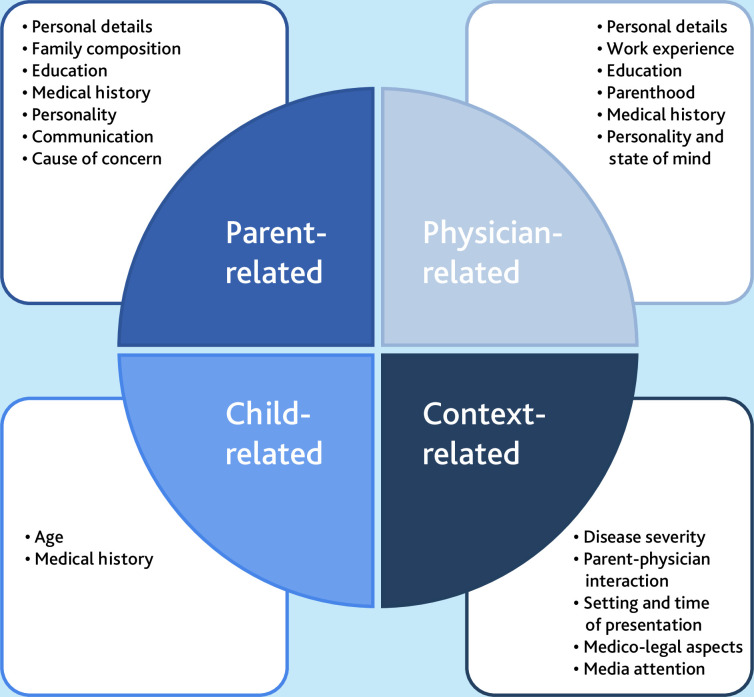
Overview of the four main categories with their different factors

Several factors have an impact on how physicians appraise their gut feeling. These can be divided into four main categories: parent-, child-, physician-, and context-related factors (Figure 1). Similar factors and themes were observed between the interviews of the paediatricians and GPs. Relevant quotes from participants are incorporated into the text below. Table 2 shows the Q-analysis of the frequency factors.

**Table 2.** Frequency of factors impacting GPs’ and paediatricians’ assessment of parental concerns (Q-analysis)

**Factor**	Participants, *n* (%)	
	**GPs (*n* = 15)**	**Paediatricians (*n* = 15)**
**Parent-related**		
Personal details	14 (93.3)	12 (80.0)
Family composition	14 (93.3)	12 (80.0)
Education	13 (86.7)	11 (73.3)
Medical history	8 (53.3)	9 (60.0)
Personality	3 (20.0)	6 (40.0)
Communication	6 (40.0)	12 (80.0)
Cause of concern	7 (46.7)	9 (60.0)
**Child-related**		
Age	9 (60.0)	7 (46.7)
Medical history	4 (26.7)	7 (46.7)
**Physician-related**		
Personal details	15 (100.0)	14 (93.3)
Work experience	15 (100.0)	14 (93.3)
Education	3 (20.0)	3 (20.0)
Parenthood	13 (86.7)	12 (80.0)
Medical history	10 (66.7)	8 (53.3)
Personality and state of mind	13 (86.7)	12 (80.0)
**Context-related**		
Disease severity	2 (13.3)	4 (26.7)
Parent–physician interaction	6 (40.0)	4 (26.7)
Setting and time of presentation	7 (46.7)	10 (66.7)
Medico-legal aspects	2 (13.3)	0 (0.0)
Media attention	3 (20.0)	0 (0.0)

#### Parent-related factors

This study identified seven parent-related factors: personal details, family composition, education, medical history, personality, communication, and the cause of concern. In terms of personal details, physicians emphasised that the gender and age of the parent are inconsequential, but factors such as language barriers and social context play a more significant role.

Regarding family composition, physicians concur that a parent’s judgement improves with the number of children they have, as experience grows. It is particularly pertinent whether the child is the parent’s first or a subsequent one:

*‘I actually think that it is mainly the number of children that plays a role and not the parents’ age. A first child is very different from a third or fourth child.’* (Paediatrician 4)

*‘If there are already older children, the parents are less likely to consult. When they do consult it is mostly a little more serious for them than if it was their first child.’* (GP I)

Moreover, as long as the person accompanying the child is involved in their care, their assessment holds value. Interestingly, paediatricians emphasised the importance of a stable support network around the family, whereas GPs attribute less significance to this factor.

Study participants universally acknowledged that a parent with a healthcare profession tends to be taken more seriously. Participants also acknowledged that education does not decisively influence their interpretation of parental anxiety; however, there are situations where it can exert an influence, such as cases involving less-educated individuals lacking basic knowledge, or highly educated individuals whose medical knowledge is overestimated:

*‘Intellect or level of education is not of great importance for us to judge a good parent. There may be an engineer sitting in front of you who cannot properly interpret his child.’* (Paediatrician 13)

GPs highlighted the advantage of possessing knowledge about the child’s and family’s background and medical history. The parents’ own medical history, in contrast to a sibling’s history, does not play a major role according to most participants. When parents recognise symptoms that were once present in one of their other children, physicians tend to be more receptive to their account:

*‘If people have experienced something serious with another child and now they recognise those symptoms, then I think we need to be more understanding* [of] *their concerns. This doesn’t mean our gut feeling will be more present.’* (Paediatrician 10)

Physicians also consider parental personality traits, encompassing aspects such as mental capacity, resilience, and insecurity. In this study, paediatricians noted that lower mental ability in parents can impact how they approach these parents, whereas GPs emphasised that parental insecurity can influence their perspective:

*‘I think parents’ ability to put things into perspective is a determining factor. The more insecure you are, the less supportive you are, and the less you can put things into perspective too.’* (GP C)

Communication style represents a crucial factor in how physicians handle parental anxiety. Physicians tend to take structured and coherent narratives more seriously. A significant component is the language barrier, which can complicate the history-taking process when parents struggle to express their concerns. Some participants acknowledged that in such cases, they may opt for additional tests sooner:

*‘Assessing that factor of parental anxiety is, of course, very important during history taking. If you can’t do that properly with that language barrier, you miss a very big piece to assess this.’* (GP M)

The parental cause of concern can often be elucidated by posing a simple question: *‘Why are you worried?’* This enquiry often yields vital additional information. In this study, several physicians conveyed that a worried parent should always be taken seriously:

*‘If parents are worried, be on your guard regardless of any other factor.’* (GP O)

#### Child-related factors

Two child-related factors were mentioned frequently during interviews: age and medical history. Physicians unanimously agreed that a child’s age influences their clinical decisions. For non-verbal children, obtaining a medical history poses greater challenges, amplifying the importance of the clinical assessment. Furthermore, younger children, especially infants, are susceptible to rapid deterioration compared with older children:

*‘The younger the child, the more vigilant you are. Especially as we know that young children give fewer signals that they are ill*.*‘* (Paediatrician 12)

A child’s medical history can affect parental concern in both directions. Parents with prior experience of severe illness in their children may be more adept at recognising serious issues, while others may be prone to heightened worry even in the case of common ailments:

*‘If you have a chronically ill child or a premature* [infant] *that does have something every so often, then of course, that plays a role. And then you have to incorporate that history into your thinking.’* (GP N)

#### Physician-related factors

Six physician-related factors were identified from the interviews: personal details, work experience, education, parenthood, medical history, and personality and state of mind.

When discussing their demographic details, all physicians concurred that their gender and ethnicity are irrelevant in the context of gut feeling. While a physician’s age correlates positively with work experience, age alone did not determine the capability to appraise a gut feeling; work experience holds far greater significance in this regard. Physicians did not suggest that those with limited experience cannot possess a valid gut feeling, but they acknowledged differences in the thought processes of more- and less-experienced physicians. Experienced doctors tend to rely on their own expertise and pattern recognition, whereas less-experienced counterparts may lean more on theoretical knowledge:

*‘I think the difference between younger and older doctors can be explained by the years of experience of the older doctors.’* (GP H)

*‘I think this is going to change during your career. In the beginning, you tend to lean more towards what you learnt in the courses and what elements you recognise from them. I think gut feeling gets a different connotation later on. I believe experience does indeed play a role there.’* (GP K)

Physicians observed that certain serious cases or past mistakes can make them more cautious in similar situations. Education is an essential concept emphasised by physicians. The quality of a physician’s training and the mentors they have had can significantly influence their future practice:

*‘When you now have a case or issue that you have heard about a few times in your training, you are going to have a gut feeling more quickly. I think this can be taught a bit, without you knowing it yourself, by the trainer or training. If you are with a physician who always says it will be nothing, you can sometimes adopt that attitude. As opposed to an anxious physician, who easily has a gut feeling, then you might adopt that more quickly. So I think training and internships can definitely have an impact as well.’* (GP L)

Parenthood of the physician themselves may exert an influence, though this was not universally agreed on by participants. Having children may not necessarily impact an individual’s gut feeling, but it tends to enhance empathy, enabling a better understanding of parental concerns in specific scenarios:

*‘It changes your empathy and you can better empathise with people, but it doesn’t change your gut feeling.’* (Paediatrician 5)

A physician’s own medical history holds limited relevance, but their own children’s medical history can play a role. For instance, they may be more adept at recognising certain symptoms due to prior exposure through their own children. Additionally, this experience may foster greater empathy for parents who have undergone similar situations:

*‘My own medical history or my partner’s or family’s doesn’t matter. More of my children’s. Whether you encounter something yourself or your grandparents, no. I think more about what you see with your children.’* (Paediatrician 15)

Personality traits are also considered, with characteristics such as cautiousness and doubtfulness making a physician more likely to act on gut feelings. Conversely, traits such as empathy, self-confidence, and active listening contribute to enhanced comfort in specific clinical scenarios. Fatigue is noted as a state of mind that can impair clear thinking, potentially lowering the threshold for experiencing a gut feeling, either more quickly or more slowly:

*‘When you have small children and haven’t slept 4–5 nights and you reach the consultations in the evening and you are tired, I do think you are less focused. The clinical examination is probably also less precise at those moments. So you pick up fewer signals. The whole card house collapses. I think fatigue and busyness has a somewhat negative impact on being alert and on your gut feeling.’* (GP C)

#### Context-related factors

Five context-related factors emerged from the discussions: disease severity, parent-physician interaction, setting and time of presentation, medico-legal aspects, and media attention. Disease severity stands out as a primary influence on gut feeling. Physicians tend to be more comfortable with common symptoms and diseases, and the frequency of consultation can impact the development of gut feelings, with consultations for recurrent, seemingly minor complaints being more susceptible to the emergence of gut feelings:

*‘Asking why they are concerned is, in my opinion, a point of departure to go along with the concerns or not. Independent of your disease severity of course, because the disease severity remains very important to us*.*‘* (Paediatrician 14)

The nature of the parent–physician interaction can also be an important factor, as familiarity with parents, as well as the frequency of their consultations, enables better assessment of their anxiety levels. GPs acknowledged an advantage in this respect due to their often long-standing relationships with families:

*‘As a GP you often care for the father, the mother, and the children, and often have done for a very long time. As such you know the family well and can better appreciate their concerns.’* (GP C)

The context of the presentation, whether in the emergency department, on-call, or during a consultation, can significantly influence gut feelings. In unfamiliar settings, such as on-call or in the emergency room, where physicians may not have prior knowledge of the patients, a greater degree of insecurity can be present. Moreover, the timing of presentation, whether during the day or at night, may also affect gut feelings:

*‘I do find it different sometimes when I’m on duty. But if you don’t know the parents like that, and don’t know the children, it sometimes takes a bit more time to properly assess the parents’ concerns.’* (GP F)

Discussions also highlighted that the increasing medico-legal nature of medicine may lead physicians to opt for more tests as a protective measure against litigation, even in cases where they do not perceive an urgent medical issue:

*‘The only thing I still care about is that defensive nature, the medico-legal aspect. I’m a bit more scared of that than I used to be anyway. Everything is on paper, everything is computerised. For example, I’m comfortable with this, but it’s still a bit odd, which is why we’re still going to do an extra examination or referral, just to be safe.’* (GP E)

Finally, heightened media attention to rare and severe diseases can generate anxiety among parents, prompting them to seek medical attention for seemingly minor complaints:

*‘Too many different media* [outlets] *where they can look for information is often not good either. Of course, in the long run they become so insecure that they no longer know what to do and call their general practitioner at the slightest abnormality.’* (GP C)

## Discussion

### Summary

This study both reaffirms established determining factors regarding physicians’ assessment of parental concerns, as described in existing literature, and uncovers family composition as a relevant but previously unreported determinant.

All physicians in this study have experienced and acted on gut feelings in their clinical practice, consistent with findings in previous studies.^3,6^ Many of these studies have researched this gut feeling of physicians regarding adult or paediatric patients, while few have focused on parental concerns.^16,17^ In this context of the physician-child-parent triad, this research has identified four categories of factors that influence physicians’ appraisals of parental concerns: parent-related, child-related, physician-related, and context-related.

### Strengths and limitations

The qualitative design of this study allowed for the identification of novel factors and determinants of gut feeling. However, the study’s limitations include a small sample size and potential selection bias in recruitment. Group interviews may have limited some participants’ expression of opinions due to peer pressure.

### Comparison with existing literature

Concerns about their children are highly prevalent among parents, and leading physicians too frequently interact with worried parents.^18^ Several studies have confirmed that the presence of parental worries can indeed influence a physician’s gut feeling.^8,10,11^ However, the exact nature of these parental concerns and their effects on physicians have not been extensively studied. This research highlights several parent-related factors that contribute, to varying degrees, to a physician’s gut feeling. A significant factor that has been found in this research is the distinction between first-time parents and those with multiple children. Participants found it logical to prioritise the concerns of parents with multiple children, believing that these parents better contextualise their concerns due to their childrearing experiences. This has been studied in the context of parent–child interactions yet hardly in the medical context.^19,20^ In this study, participants did not consider the influence of a parent’s gender or age on their gut feeling to be a decisive factor. For gender, this aligns with previous research that suggests that both fathers and mothers can equally recognise their child’s needs.^21^ For age, unlike in this study, it is described elsewhere in the literature that young parents tend to worry more frequently and consult for milder or benign issues.^18,22^ In this study, the presence or absence of a stable social network was found to impact parental concerns, potentially due to the inability of individuals without such a network to validate their worries through discussion with others. This finding aligns with previous research, which observed that single parents tend to have more concerns.^18^ Similarly, the reason behind parental concerns, as highlighted in this study, is also regarded as important in other research.^23^ Additionally, the dissimilarity between a current illness and a parent’s previous experience has been identified as a crucial contextual factor, both in this and previous research.^8^ This study has shown that effective communication is a key element in understanding concerns, with previous research showing language barriers to potentially hasten the emergence of gut feelings.^24^

Age and medical history are two child-related factors discussed in this study. Although physicians noted that a younger child’s age can influence their gut feeling due to communication challenges and unclear symptoms, existing research does not consistently support this. For example, in acute cough and respiratory tract infections, age did not correlate with gut feeling.^9^ However, study participants identified 3 months as a crucial age limit, below which gut feeling is more significant. The physicians also stated that a child’s medical history may impact their gut feeling, like studies in adult patients with cancer, which have suggested that a patient’s medical history can trigger gut feelings.^25^ While specific studies on a child’s medical history are limited, it is reasonable to assume that comorbidities in children could influence gut feelings.^26^

Several physician-related factors discussed in the focus group interviews align with existing literature. It is the character of the physician, rather than their gender, that appears to have a significant impact on gut feeling, as observed in both this study and existing literature.^27^ Age and experience were also thought to influence gut feeling, as in previous research that has shown that doctors aged >50 years and those with >15 years’ experience have a more accurate gut feeling.^25^ Experience is consistently recognised as a critical factor for a physician developing and trusting their own gut feeling, with participants in this study feeling that less-experienced physicians often engage in more analytical reasoning.^2,4,12,25,27,28^ This study, alongside existing literature, indicates that medical knowledge plays a role in that physicians cannot worry about what they do not know; however, a lack of knowledge can also lead to concerns more quickly.^4,27^ In this study, personality traits such as empathy and self-confidence were identified as important in the context of gut feeling. Other studies have found that high empathy scores in physicians were associated with more frequent gut feelings.^28–30^ The majority of this study’s participants reported that parenthood enhanced their empathetic abilities and deepened their understanding of parents’ perspectives. This observation aligns with a study that found that paediatric residents also experienced an improved understanding of parents after becoming parents themselves.^31^ However, it is noteworthy that heightened empathic feelings do not necessarily equate to superior perspective-taking skills.^32,33^ In addition to the influence of parenthood, this study and existing literature indicate that personal and family-related events can impact a physician’s gut feeling.^34^ This study found that high workload and fatigue can negatively impact empathy, which is consistent with previous research.^27,35^ Published data further demonstrate that extended working hours and sleep deprivation contribute to diminished attention, reduced vigilance, decreased empathy, impaired cognitive performance, delayed reaction time, and an increased likelihood of medical errors.^35–38^ Given that several of these factors are pivotal in the context of gut feeling, it is reasonable to infer that fatigue can impact this diagnostic intuition. Furthermore, self-confidence emerged as a notable characteristic in discussions in this study. Individuals with lower self-confidence may be less inclined to rely on their sense of reassurance, as supported by existing literature.^2^ Additionally, this study concurs that the manner in which a physician copes with uncertainty has repercussions on the presence of gut feelings.^4^ The implications of medical errors were also addressed. Virtually every physician encounters medical errors, either directly or indirectly, during their professional career, which can have lasting effects, as indicated by the study participants. These errors, according to research, not only impact patients but also have repercussions for physicians and the healthcare system.^39^ Physicians who make mistakes often experience emotions such as guilt and anxiety, which can lead to a reduction in empathy according to multiple studies.^39,40^ Some physicians, similar to certain participants in this study, reported that their reluctance to take action is diminished after a medical error.^41^ Conversely, another study suggested that medical errors could also have a positive impact, fostering increased confidence, assertiveness, and improved relationships with colleagues.^42^ Nevertheless, whether these experiences directly influence gut feeling remains an unexplored area in the existing literature.

Finally, there are several factors that are context-related. According to participants of this study, disease severity seems to have a major impact on gut feeling, a finding that is corroborated by existing literature.^8^ Study participants and literature state that gut feelings are more readily experienced when children are more unwell.^8–10^ Physician–patient relationship also emerged as an important factor affecting gut feeling in this study. Knowing a parent well enables physicians to better understand the parental behaviours and to gauge their concerns more accurately.^25,27^ Additionally, participants in this study, as in other studies, agreed that knowledge not only of a patient’s health but also of how the family deals with health issues can impact gut feeling.^22,27^ The timing of a patient’s presentation, as observed in this study, can affect gut feelings, with night-time presentations potentially leading to heightened gut feelings.^27^

While this study highlights the importance of gut feelings, a concept that is often associated with an intuitive, non-analytic process, many of the identified factors influencing these feelings — such as family composition, sibling medical history, and physician experience — are relatively concrete and may represent rapid, subconscious integration of multiple data points rather than purely intuitive judgements. This aligns with perspectives from existing literature suggesting that gut feeling may be underpinned by complex cognitive processes rather than being entirely instinctual.^43^ Moreover, there is a potential risk of legitimising cognitive biases through reliance on gut feelings. For instance, subjective impressions related to parental education levels or other sociodemographic factors could inadvertently lead to inequitable or inaccurate clinical decisions. Recognising these risks, this study underscores the need for heightened awareness of how non-analytic factors, including potential biases, shape decision making in triadic consultations involving children, parents, and physicians. Physicians should remain aware of the interplay between intuitive, emotional, and analytic processes in their clinical reasoning while addressing mechanisms to mitigate bias.^44^

### Implications for research and practice

This qualitative study has identified four major groups of factors that influence physicians’ assessments of parental concerns: parent-related, child-related, physician-related, and context-related. While some factors, such as experience and physician–patient relationship, were already known to be important, new determinants, such as having multiple children and physician fatigue, have been uncovered. Future research should quantitatively assess the extent to which these identified factors contribute to gut feeling appraisal. Furthermore, exploring parents’ and children’s perspectives and conducting quantitative research could provide valuable insights into how physicians consider concerns from these stakeholders. This information can inform medical practice, helping physicians better address the needs of patients and parents alike.

## Data

The full dataset of this study can be obtained by readers at request from the corresponding author.
